# MBC and ECBL libraries: outstanding tools for drug discovery

**DOI:** 10.3389/fphar.2023.1244317

**Published:** 2023-08-11

**Authors:** Tiziana Ginex, Enrique Madruga, Ana Martinez, Carmen Gil

**Affiliations:** ^1^ Centro de Investigaciones Biológicas “Margarita Salas” (CIB-CSIC), Madrid, Spain; ^2^ Centro de Investigación Biomédica en Red en Enfermedades Neurodegenerativas (CIBERNED), Instituto de Salud Carlos III, Madrid, Spain

**Keywords:** drug discovery, chemical libraries, virtual screening, molecular diversity, chemical space

## Abstract

Chemical libraries have become of utmost importance to boost drug discovery processes. It is widely accepted that the quality of a chemical library depends, among others, on its availability and chemical diversity which help in rising the chances of finding good hits. In this regard, our group has developed a source for useful chemicals named Medicinal and Biological Chemistry (MBC) library. It originates from more than 30 years of experience in drug design and discovery of our research group and has successfully provided effective hits for neurological, neurodegenerative and infectious diseases. Moreover, in the last years, the European research infrastructure for chemical biology EU-OPENSCREEN has generated the European Chemical Biology library (ECBL) to be used as a source of hits for drug discovery. Here we present and discuss the updated version of the MBC library (MBC v.2022), enriched with new scaffolds and containing more than 2,500 compounds together with ECBL that collects about 100,000 small molecules. To properly address the improved potentialities of the new version of our MBC library in drug discovery, up to 44 among physicochemical and pharmaceutical properties have been calculated and compared with those of other well-known publicly available libraries. For comparison, we have used ZINC20, DrugBank, ChEMBL library, ECBL and NuBBE along with an approved drug library. Final results allowed to confirm the competitive chemical space covered by MBC v.2022 and ECBL together with suitable drug-like properties. In all, we can affirm that these two libraries represent an interesting source of new hits for drug discovery.

## 1 Introduction

Virtual high throughput screening (vHTS) represents a gold standard in modern drug discovery workflows especially for Pharma and Biotech companies (Subramaniam et al., 2008; Tanrikulu et al., 2013). Integration and complementation of *in silico* tools to classical HTS has boosted the capability of rapidly exploring a wider chemical space for the effective identification of new hits with indirect beneficial effects also on further steps of drug discovery as hit-to-lead optimization (Bajorath, 2002). The *in silico* techniques generally applied in this context are based on a common principle that is the accurate and effective assessment of the chemical complementarity between the protein target of interest and small molecules. In the case of ligand-based techniques as the mainstream QSAR-based (Neves et al., 2018) or pharmacophore-based (Kim et al., 2010) virtual screening, preliminary and well-curated experimental data for representative chemical scaffolds are needed. These data in conjunction with a proper selection of relevant atomic or molecular descriptors for the compiled list of active compounds are then used to guide the search for new compounds. In the case of structure-based approaches as docking-based virtual screening, each compound in the chemical library is screened for its binding affinity toward a given target by using properly tuned scoring functions (Neves et al., 2021). Best scored molecular candidates from both approaches can be used for preliminary proof-of-concept. In case active compounds are found, they can go to further structural optimization with the ultimate goal of maximizing both biological effect and pharmacokinetic properties.

One strategy that can be envisaged to rise the chances to find effective compounds is represented by the use of big non-enumerated libraries. Nowadays, we are assisting to the growing of huge chemical libraries with an average number of compounds from 10^10^ to 10^20^ (Nicolaou et al., 2016). Despite they enable access to an impressive large chemical space, a common bottleneck is still represented here by the *in silico* tools since a complete structure-based screening of such huge libraries would require unaffordable computational costs and time. Besides, such huge chemical libraries have a number of compounds with properties far from being optimal to be considered as hits. To overcome these limitations, machine learning models from the implementation of Bayesian optimization algorithms for docking-based virtual screening would significantly reduce the computing time making the screening of large chemical library possible (Graff et al., 2021).

Another more feasible possibility could be represented by the use of focused chemical libraries. These kinds of libraries are generally small, drug-like collections and come from a focused enumeration of compounds acting on specific targets as kinases (Kéri et al., 2005), protein-protein interactions (PPi) (Sperandio et al., 2010), G-coupled receptors (Jimonet and Jäger, 2004) among others. The use of such small libraries indeed would allow to shorten the computing time. Best candidates from preliminary screening can be then used to setup *ad hoc* optimization strategies aimed at improving activity toward a specific protein target of interest (Balakin et al., 2006; Mayr and Bojanic, 2009). The advantage of using quality-focused libraries resides in the fact that properties for compounds are already partially optimized. Moreover, a clear linkage between structure and biological activity is also guaranteed.

**FIGURE 1:**
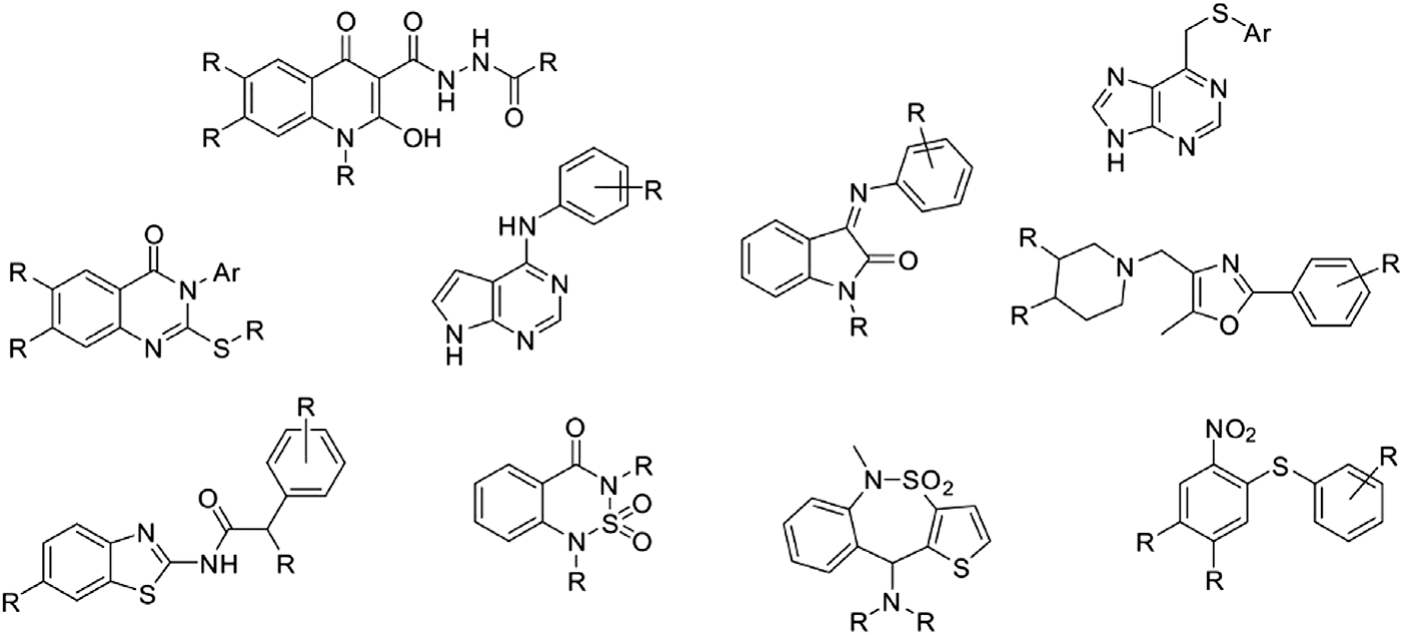
Chemical scaffolds for the first 10 most populated clusters of the MBC v.2022 library.

Our laboratory has developed an in-house quality-focused chemical library named Medicinal and Biological Chemistry (MBC) library, that condensates more than 30 years of medicinal chemistry research in our group. It contains compounds with a standard chemical purity of at least 95% by HPLC and is available both electronically and physically upon request. Since its first publication in 2017 (Sebastián-Pérez et al., 2017), the MBC library has grown significantly reaching a total of 2,577 curated compounds with annotated data about activity and purity. Compounds of the first version of the MBC library (MBC v.2016) have been designed mainly as potential drugs for neurological and neurodegenerative diseases but can be also used as a useful reservoir for the treatment of other diseases. The actual version (MBC v.2022) has been enriched by novel chemical series that have been developed for different targets as those responsible for neglected or infectious diseases, among others. The most representative chemical families of the new MBC v.2022 library are reported in Figure 1.

To validate the quality of the updated MBC library, up to 44 physicochemical and pharmaceutical properties have been calculated for all the compounds with particular attention to drug-likeness properties. The original version of the library (MBC v.2016) has been compared with the new one (MBC v.2022) to quantify the level of improvement of the new version. To exclude structural redundancy, special attention has been deserved to the analysis of the structure (i.e., Tanimoto similarity) and substructure (i.e., Bemis-Murcko algorithm) variability. Finally, to provide a wider perspective, the MBC v.2022 library has been also compared with others well-known chemical libraries such as ZINC20 (Irwin et al., 2020), DrugBank (Wishart et al., 2018), ChEMBL (Mendez et al., 2019), NuBBE (Saldívar-González et al., 2019) and the Approved drug library from Selleck Chemicals together with the European chemical biology library (ECBL) (Horvath et al., 2014). This last library was assembled by the European research infrastructure consortium (ERIC) for chemical biology named EU-OPENSCREEN (EU-OS) (Frank, 2014; Brennecke et al., 2019). This ERIC integrates high-capacity screening platforms throughout Europe with the ECBL (Horvath et al., 2014) and medicinal chemistry expert laboratories making available new hit discoveries for a selected target and the *hit-to-lead* optimization.

## 2 Materials and methods

### 2.1 Database’s collection

The MBC v.2016 (1,096 compounds) (Sebastián-Pérez et al., 2017), MBC v.2022 (2,577 compounds), ECBL (101,021 compounds; https://www.eu-openscreen.eu/services/database.html), ZINC20 (Irwin et al., 2020) (10,723,360 compounds; https://files.docking.org/zinc20-ML/), DrugBank v.5.0 (Wishart et al., 2018) (10,981 compounds; https://go.drugbank.com/releases/latest), ChEMBL v.31 (Mendez et al., 2019) (1,908,325 compounds; https://chembl.gitbook.io/chembl-interface-documentation/downloads), NuBBE (Saldívar-González et al., 2019) (2,223 compounds; https://nubbe.iq.unesp.br/portal/nubbe-search.html) and Approved drug library (3,104 compounds; https://www.selleckchem.com/screening/fda-approved-drug-library.html) databases downloaded from their websites in September 2022 were considered for comparison. For comparative purposes, focused subsets of the freely available databases ZINC20, DrugBank, ChEMBL were considered. Briefly, the in-stock drug-like subset was used in case of the ZINC20 database. Regarding the ChEMBL database, only small molecules was selected, discarding other entries (as antibodies or enzymes) out of the scope of this study. Finally, for the DrugBank library, biotechnology products were ignored. All the material and data produced for this study along with the python scripts used to reproduce all the graphics are available at https://doi.org/10.5281/zenodo.8212104.

### 2.2 Database’s preparation

For all the databases, the 3D structures were generated with the LigPrep module of the Schrödinger suite (Schrödinger, 2022: LigPrep, Schrödinger, LLC, New York, NY, 2022) in accordance with our previous study (Sebastián-Pérez et al., 2017). In brief, molecules were protonated according to the protonation state at physiological pH. All counterions were removed and no tautomers were generated. Finally, stereochemistry was retained according to the original entries. In-house python scripts along with the pandas-1.4.4, matplotlib-3.6.0 and seaborn-0.12.0 modules of python3 were used to produce all the graphics and statistics reported in this study.

### 2.3 Properties’ calculation

Pharmaceutically relevant principal descriptors for all the compounds (see Table 1) were calculated using the QikProp v.6.8 (Schrödinger Release, 2022: QikProp, Schrödinger, LLC, New York, NY, 2022). As stated in the manual, QikProp is unable to calculate properties for not neutralizable quaternary ammonium compounds, so we were forced to exclude these compounds from all the analyzed databases. For this reason, 7.39%, 3.14%, 0.20%, 4.13%, 0.61%, 2.97%, 4.62%, and 0.14% of the prepared compounds respectively from MBC v.2016, MBC v.2022, ECBL, DrugBank, ZINC, ChEMBL, approved drug library and NuBBE were excluded. The probability of a false readout in a screening assay was determined by HitDexter3.0 (Stork et al., 2020; Stork et al., 2021). Similarly, 3.61% and 0.15% of MBC v.2022 and ECBL were not able to be processed. With regard to Veber and Ghose filters, both were calculated with RDKit (Landrum, 2016; Bento et al., 2020). The corresponding measurements and thresholds can be found elsewhere (Ghose et al., 1999; Veber et al., 2002).

**TABLE 1:** Quantitative distributions for the most relevant pharmacokinetic properties of the MBC and ECBL libraries calculated with QikProp.

Property	Intervals	MBC v.2016	MBC v.2022	ECBL
Lipinski’s rule of 5	0 violations	85.1%	85.3%	98.7%
	1 violation	13.4%	12.0%	1.0%
	2 violations	1.5%	2.3%	0.2%
	3 violations	0.0%	0.4%	0.1%
	4 violations	0.0%	0.0%	<0.1%
Jorgensen’s rule of 3	0 violations	76.0%	76.1%	92.4%
	1 violation	23.5%	23.1%	7.3%
	2 violations	0.5%	0.8%	0.2%
	3 violations	0.0%	0.0%	<0.1%
Veber filter	Meet the criteria	96.4%	95.1%	95.4%
Ghose filter	Meet the criteria	85.8%	85.8%	94.9%
MW (Da)	0–200	9.6%	5.9%	0.2%
	201–300	36.5%	34.4%	21.0%
	301–400	39.2%	42.0%	64.0%
	401–500	12.9%	14.4%	14.1%
	>500	1.8%	3.3%	0.7%
Nr. of rotatable bonds	0–5	82.1%	79.5%	66.9%
	6–10	7.1%	17.1%	27.0%
	>10	0.8%	3.4%	6.1%
donorHB (HBD)	≤5	99.9%	99.8%	99.9%
	>5	0.1%)	0.2%	0.1%
accptHB (HBA)	≤10	99.4%	98.0%	94.0%
	>10	0.6%	2.0%	6.0%
QPlogP_o/w_	≤5	91.0%	87.5%	99.5%
	>5	9.0%	12.5%	0.5%
QPlogS	−12.0/−7.0	11.6%	6.7%	0.4%
	−6.9/−3.0	73.3%	82,9%	67.0%
	−2.9/2.0	15.1%	10.4%	32.6%
QPlogBB	−9.0/−5.0	0.0%	0.0%	<0.1%
	−4.0/−1.0	61.2%	62.0%	20.2%
	−0.9/2.0	38.8%	38.0%	79.8%
Human oral absorption in GI	0%–50%	1.9%	2.1%	0.7%
	51%–75%	9.6%	8.5%	12.5%
	76%–100%	88.5%	89.4%	86.8%
Probability of highly promiscuous activities in target-based assays	0.00–0.50	-	93.2%	99.3%
	0.51–0.75	-	0.7%	0.1%
	0.76–1.00	-	2.6%	0.4%

### 2.4 Structure similarity analysis

A wide chemical space as a result of a large chemical diversity in chemical libraries is of utmost importance in rising the chances of finding effective and thus promising hits in drug discovery (Gerry and Schreiber, 2018). In this scenario, the Tanimoto coefficient has been routinely used to evaluate chemical similarity or variability (Bajusz et al., 2015). The Tanimoto coefficient (*T*_*c*_) between two points, *a* and *b*, with *k* dimensions is calculated according to Eq. 1

$$T_{c}=\frac{\sum_{j=1}^{k}a_{j}\times b_{j}}{\left(\sum_{j=1}^{k}a_{j}^{2}+\sum_{j=1}^{k}b_{j}^{2}-\sum_{j=1}^{k}a_{j}\times b_{j}\right)}$$ (1)

The pairwise comparison of fingerprints—one for the query and one for the target structure - allows to obtain the global similarity between two molecules (*T*_*c*_) which can vary between 0.0 (no similarity) and 1.0 (maximum similarity or identity).

Tanimoto similarity matrixes for the MBC v.2016, MBC v.2022 and ECBL libraries were generated with RDKit (Landrum, 2016; Bento et al., 2020). Accordingly, the SMILES codes for each molecule of the previously cited datasets were first converted in RDKit molecules and molecular fingerprints were thus calculated. Comparison of the so generated RDKit fingerprints allowed to generate a NxM matrix whose dimensions depends on the length of the analyzed database. Accordingly, 1,096 × 1,096, 2,577 × 2,577 and 101,021 × 101,021 Tanimoto matrixes were generated respectively for the MBC v.2016, MBC v.2022 and ECBL libraries and plotted (Figure 4). SMILES codes for compounds bearing a quaternary ammonium - for which QikProp was unable to calculate properties - were retained for this analysis.

### 2.5 Substructure similarity analysis

A quite common scaffold representation is the Murcko framework proposed by Bemis and Murcko (Hu et al., 2016). Given a query molecule, the method employs a systematic dissection into four parts: ring systems, linkers, side chains, and the Murcko framework that is the union of ring systems and linkers in a molecule. The information obtained by this analysis can be used for different purpose as, for instance, database enumeration. In this work, the Bemis-Murcko scaffolds have been calculated for each input RDKit molecule by using the ChemAxon Bemis-Murcko node of the KNIME platform (https://www.knime.com/knime-analytics-platform). The resulted scaffolds were finally clustered according to their canonical SMILES codes.

## 3 Results and discussion

Successful screening projects begin with the selection of appropriate chemical libraries in terms of size, quality, and chemical diversity. Unlike ultra-large libraries, which are computationally expensive to use, quality-focused chemical libraries represent a useful source of chemical entities enriched with active chemotypes that may be designed to efficiently combine chemical diversity along with a significant reduction of the computational resources eventually required for a screening campaign. Moreover, these libraries may be built according to drug likeness properties (e.g., ADME/Tox properties), offering promising starting-points that can accelerate hits finding and *hit-to-lead* protocols (Gong et al., 2017).

### 3.1 The Medicinal and Biological Chemistry (MBC) library from 2016 to 2022

**FIGURE 2:**
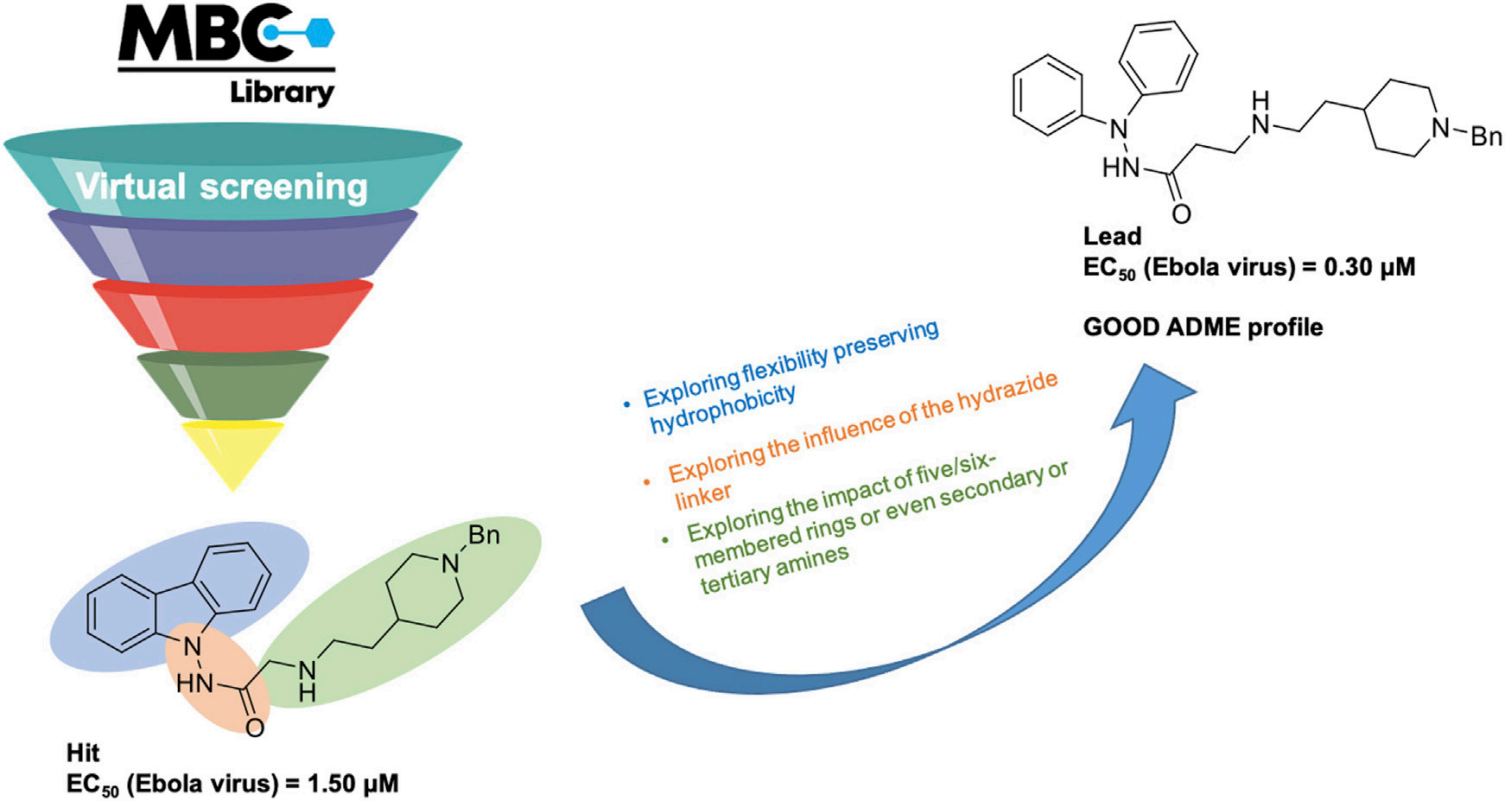
Hit-to-lead approach followed around the carbazole hit identified as Ebola virus entry inhibitor by virtual screening using the MBC library (Garcia-Rubia et al., 2023).

The MBC library has originated from more than 30 years of experience in drug discovery of our research group. It has been conceived as a collection of focused sets of chemical probes with common therapeutic profiles mostly in the field of neurodegenerative and infectious diseases as such as Alzheimer’s and Parkinson’s diseases, amyotrophic lateral sclerosis (ALS), schistosomiasis, and leishmaniasis, among others. It represents a source of fully accessible, ready-to-use compounds with proved efficacy. The library has been growing from 1,096 compounds in 2016 to 2,577 compounds in 2022 with a significant exploitation in the field of infectious diseases. The utility of the MBC library to initiate drug discovery programs is reflected mainly in the neurodegenerative and anti-infective fields. Particularly, successful families of CK1 inhibitors with a benzothiazole core (Salado et al., 2014; Martínez-González et al., 2020) and CDC7 inhibitors with a 6-mercaptopurine scaffold (Rojas-Prats et al., 2021) useful for ALS were developed till the *in vivo* proof of concept after initial hit identification using the MBC library as reported. Very recently new mitophagy modulators having chemically diverse scaffolds were also discovered (Maestro et al., 2023). In the anti-infective field it is remarkable the discovery of *N′*-phenylacetohydrazide derivatives as potent Ebola virus entry inhibitors (Garcia-Rubia et al., 2023) starting with a carbazole hit identified from the MBC library (Lasala et al., 2021) (Figure 2). The increased potentialities of the new version of our MBC library have been addressed here and compared with the previous version (Sebastián-Pérez et al., 2017).

### 3.2 The European Chemical Biology Library (ECBL)

The selected EU-OPENSCREEN (EU-OS) compound collection is centrally stored and managed at the EU-OS laboratory facility on the Research-Campus Berlin-Buch (Germany). All compound structures and primary screening data will be published in the open-access European Chemical Biology Database, where they are made available to a wide scientific audience. The European research infrastructure EU-OS collaboratively develops novel molecular tool compounds and early therapeutic candidate molecules together with external users from various disciplines of the life sciences. Access to the EU-OS resources is open to researchers from academia and industry from countries inside and outside of the European Union. The current version (v.2022) of the ECBL integrates 101,021 available, ready-to-use compounds with unbiased chemical diversity, designed by five renowned academic computational chemistry groups. To maximize the coverage of chemical space, criteria followed by these groups in the molecules selection were completely different but chemical stability, drug-likeness criteria, and practical availability were pursued in all the cases (Horvath et al., 2014). Recently, this library have started to be used providing valuable hits to fight against COVID-19 (Kuzikov et al., 2021; Schuller et al., 2021).

### 3.3 Comparative analysis

**FIGURE 3:**
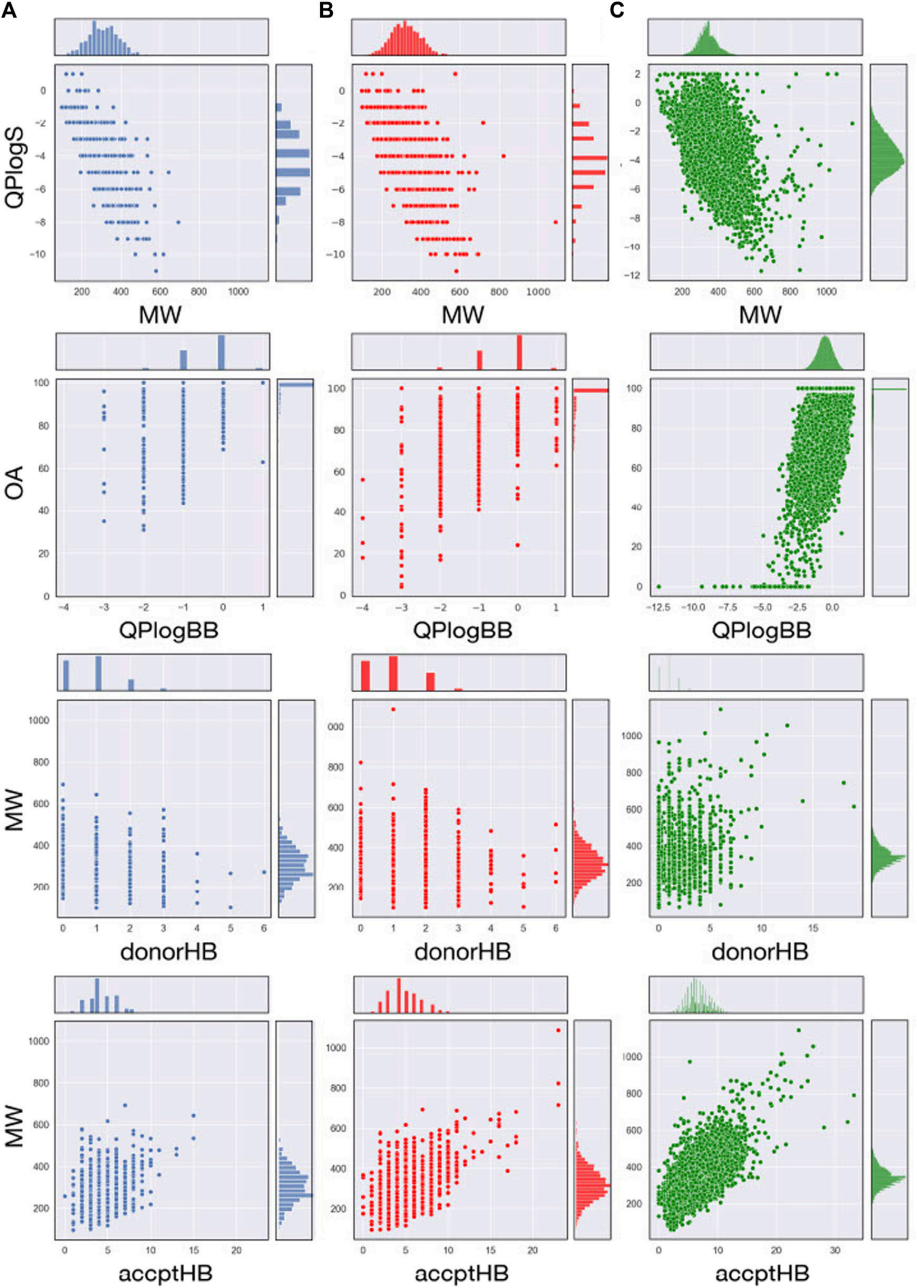
Join scatter and distribution plots of the main principal descriptors and properties calculated with QikProp for the MBC v.2016 (**A**; in blue), MBC v.2022 (**B**; in red) and for ECBL (**C**; in green) libraries.

The most relevant physicochemical and pharmaceutical properties for each of the compound of the MBC library have been predicted with the QikProp module of Schrödinger. Quantitative distributions for the most relevant pharmacokinetic properties of the two versions of the MBCs library are reported in Table 1. For a comparative description, relative dispersion and distribution for some salient pharmacokinetics (PK) properties as molecular weight (MW), predicted octanol/water logP (QP logP), predicted logS (QP logS), hydrogen-bond donor (HBD), hydrogen-bond acceptor (HBA), predicted blood-brain barrier (BBB) permeability (QP logBB) and human oral absorption (OA) are showed in Figure 3.

A traditional method to evaluate drug likeness is represented by the Lipinski’s Rule of Five (Lipinski et al., 2001; Lipinski, 2004). In line with the previous version of the library, the 97.3% (85.3% with 0 violations and 12.0% with 1 violation) of the compounds in the MBC v.2022 have less than 2 violations of the Lipinski’s Rule of Five. Less than 3% have more than 2 violations. The Jorgensen’s Rule of Three (Lionta et al., 2014) is another widely followed rule for lead like properties and states that the aqueous solubility measured as logS should be greater than −5.7, the apparent Caco-2 cell permeability should be faster than 22 nm/s, and the number of primary metabolites should be less than 7. In both versions of the MBC library, the majority of the compounds (76.0%) have no violations with the 23% showing only 1 violation.

Molecular flexibility, number of hydrogen-bonding donor/acceptor groups and molecular weight are critical parameters for drug likeness. In the MBC library, most of compounds (90.8% of the MBC v.2022 and 88.6 of the MBC v.2016 library) have a MW between the recommended interval of 200–500 Da (see Table 1; Figure 3). More than 90% of the MBC compounds have up to 10 rotatable bonds. More than 98% have less than 5 hydrogen-bond donor and less than 10 hydrogen-bond acceptor groups (see Figure 3).

Lipophilicity (Ginex et al., 2019) and thus solubility (Bergström and Larsson, 2018) have a great impact on the pharmacokinetic profile of a potential drug. Most of the compounds of our MBC library have suitable lipophilicity and solubility predicted values (more than 80% with QP logP_o/w_ below 5 and QP logS between −6.5 and 0.5; see Table 1; Figure 3). This is also reflected in a good BBB predicted permeability with about 60% of compounds with QPlogBB values between −1 and −4 (see Table 1). Here, a close look at the distributions for the QPlogBB values reported in Figure 3 allows to see that most of the compounds specifically fall within 0 and −1. Finally, more than 95% of the compounds have a predicted oral absorption (hOA) rate in the gastrointestinal (GI) tract higher than 50% (see Table 1; Figure 3). Moreover, potentially promiscuous compounds should be carefully treated and analyzed in order to avoid false-positive results. There is a wide range of strategies to afford this, from classical substructure detection [e.g., Pan Assay Interference Compounds (PAINS) filter (Baell and Holloway, 2010)] to more refined machine learning methodologies (Blaschke et al., 2019). In this sense, the probability of triggering a positive result in a target-based screening, understood as a false positive due to the chemical promiscuity of the molecule, was calculated here using HitDexter 3.0 server (https://nerdd.univie.ac.at/hitdexter3), a machine learning approach that shows how the vast majority of the MBC library (93.2%) avoid this alert.

The analysis of the most relevant physicochemical and pharmacokinetic properties for ECBL has been reported in Table 1 and Figure 3C. In brief, the 98.7% and the 92.4% of the compounds have no violations of respectively the Lipinski’s Rule of Five and Jorgensen Rule of Three which is globally indicative of the high pharmaceutical relevance of the dataset. As demonstrated by the data in Table 1 and plots in Figure 3C, this dataset guarantees an excellent coverage of the drug-like chemical space with MW lower than 600 Da, a number of rotatable bonds lower than 10, less than 5 hydrogen-bond donor and less than 10 hydrogen-bond acceptor atoms. QPlogBB values fall in the interval of 2 and −4 (see Table 1), with the majority of the compounds within 1 and −2.5 (see Figure 3). The good characterization, data curation and immediate availability of the compounds of the ECBL make it also a good reservoir of potential hits. Finally, the HitDexter program shows how the 99.3% of this library present low probability of trigger a false-positive readout in target-based assays.

### 3.4 Tanimoto similarity

Beside physicochemical and PK properties, a wide chemical variability or diversity is also a pivotal feature since it could influence the success rate of a screening protocol (López-Vallejo et al., 2012). The use of small-sized libraries with low chemical variability and high structural redundancy could in fact reduce the possibility to find useful hits.

**FIGURE 4:**
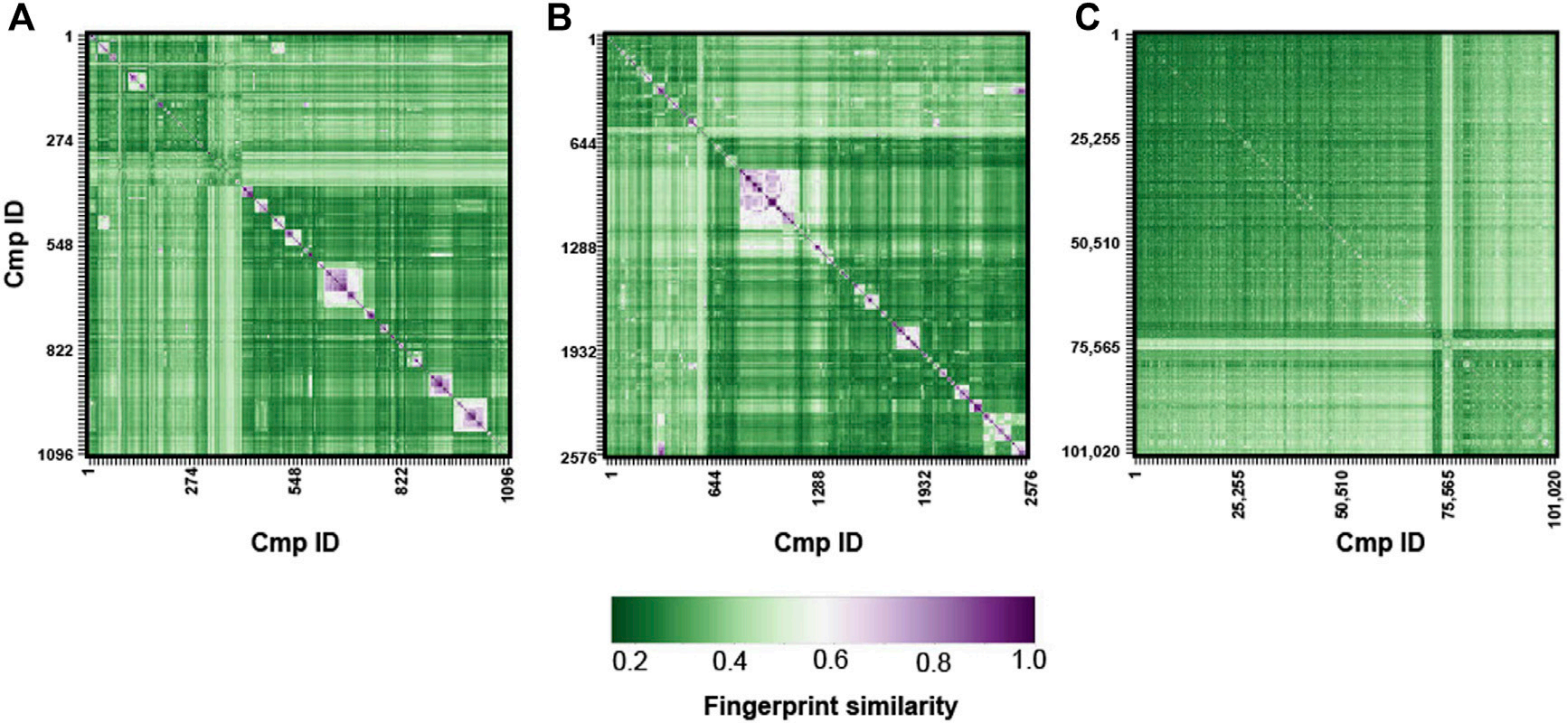
Tanimoto-based fingerprint similarity analysis for the MBC v.2016 **(A)**, MBC v.2022 **(B)** and ECBL **(C)** libraries.

In this regard, the Tanimoto metric has been widely used to evaluate molecule similarity thus it represents a valid way to measure the qualitative chemical variability of a compound’s library (Sankara Rao et al., 2011; Bajusz et al., 2015; Xia and Yan, 2017). The chemical variability of our MBC library has been subjected to Tanimoto-based fingerprint similarity analysis (see Figure 4). With the exception of a small cluster of *structurally-related,* relatively similar compounds with values among 0.5 and 0.7 (white/pink square in the similarity matrix for MBC v.2016 and v.2022), a clear predominance of fingerprint values lower than 0.5 is generally observed thus confirming the suitable chemical diversity of our library. Regarding the ECBL, no similarity clusters were found as shown by the low values of the Tanimoto coefficients. This once again highlights the valuable chemical diversity of this library.

### 3.5 Scaffold clustering

**FIGURE 5:**
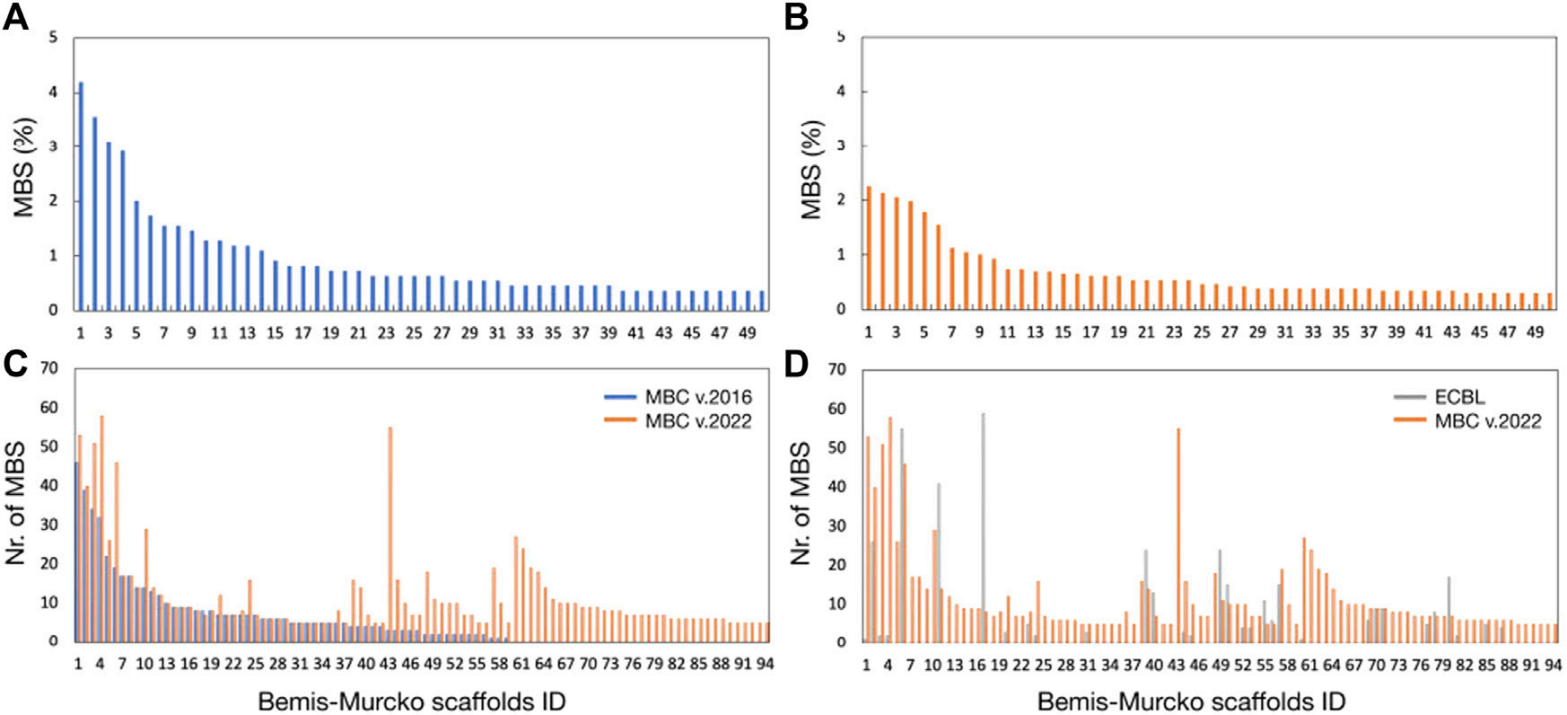
Bemis-Murcko scaffold distribution (%) for the MBC v.2016 **(A)** and the MBC v.2022 **(B)**. Analysis of the common scaffolds between MBC v.2022 and MBC v.2016 or ECBL is reported respectively in **(C**, **D)**. For clarity, only the scaffolds with a population >5 compounds have been shown.

Bemis and Murcko outlined a popular method for deriving scaffolds from molecules by removing side chain atoms (Bemis and Murcko, 1996). Widely speaking, the Bemis-Murcko framework algorithm represents an effective indicator of the chemical diversity of the chemical libraries. The ChemAxon extended version of the Bemis-Murcko framework algorithm implemented in KNIME (Hu et al., 2016) has been used here to perform scaffold clustering to check the chemical diversity of the MBC v.2022 and the ECBL. More details about the procedure can be found in the *Materials & Methods* section. For comparative purposes, the old version of the MBC library (v.2016) has been also included in the analysis (see Figure 5).

**FIGURE 6:**
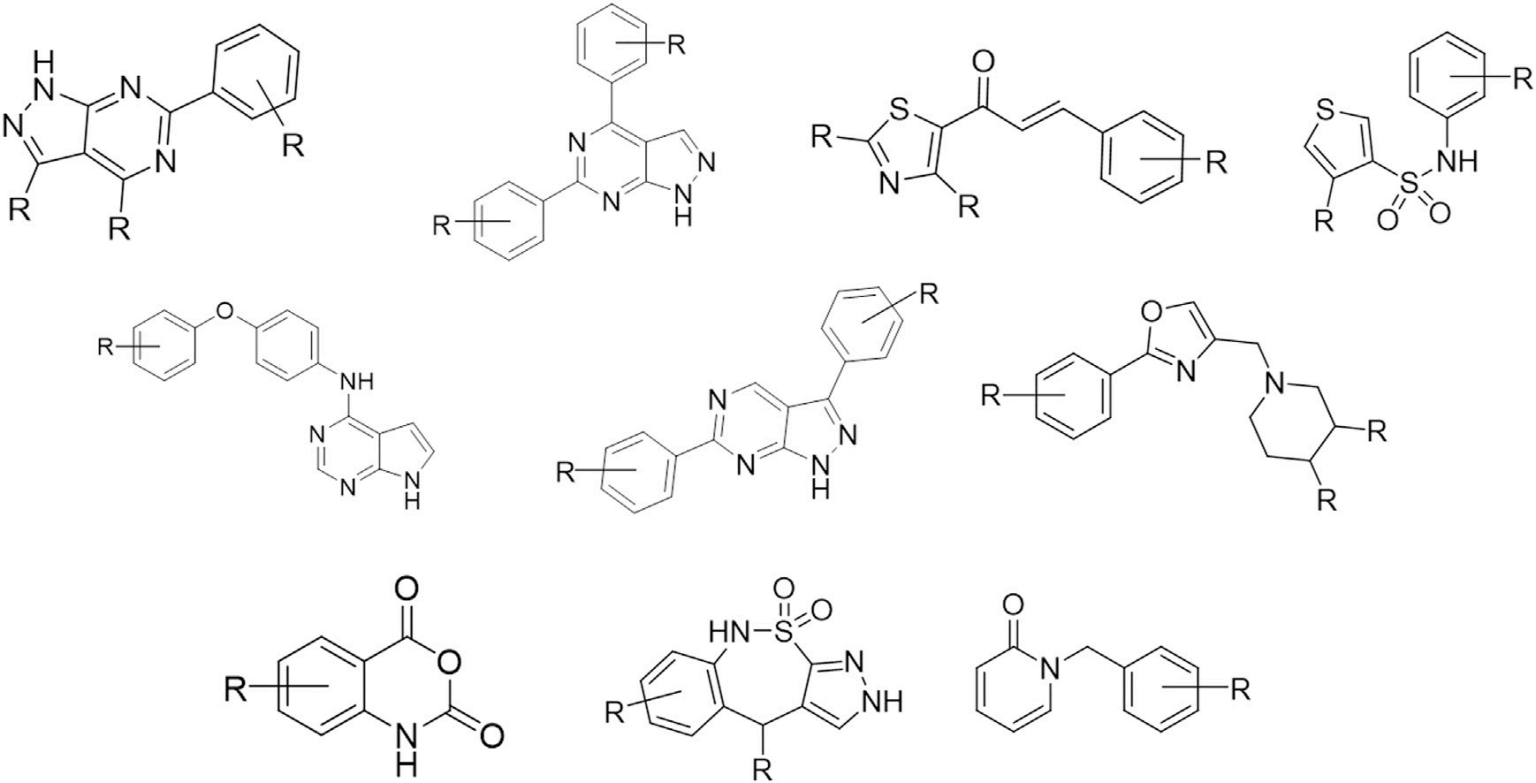
Most representative new scaffolds from the MBC v.2022 with respect to the MBC v.2016.

A total of 465 and 1123 Bemis-Murcko scaffolds have been found respectively for the MBC v.2016 and v.2022 libraries thus confirming the enrichment in chemical variability of the new version. The most representative new scaffolds from MBC v.2022 with respect to the v.2016 are depicted in Figure 6. As observed in Figures 5A,B, a high level of chemical diversity generally characterizes the MBC v.2022 database with only 2 Bemis-Murcko scaffolds having a population above the 2% of the structures present in the MBC v.2022 database, showing that the vast majority of the compounds are distributed over different chemotyes (Figure 5B). Library expansion could be due to two possible factors as 1) the enrichment of already present scaffolds by means of further enumeration or 2) the introduction of totally new chemical entities. In case of the MBC library, analysis of the common scaffolds (see Figure 5C) between the two versions allowed to see that the library expansion generally came from the introduction of new chemical species with a limited enumeration of the scaffolds already present in the previous version (see Bemis-Murcko scaffolds IDs 1-59 in Figure 5C). Finally, the uniqueness of the chemical scaffolds collected in the MBC v.2022 library has been evaluated with respect to the ECBL. On a total of 94 scaffolds with population higher that 5 compounds, there are 62 unique structures in the MBC v.2022 library (see Figure 5D) and only 32 are shared with the ECBL.

### 3.6 Comparison with other libraries

The physicochemical and drug-like properties of the MBC library have been also compared with those of some publicly available chemical databases as ZINC, DrugBank, ChEMBL, NuBBE and approved drug library from Selleck Chemicals (henceforth referred to as Approved drug library). ZINC (Irwin et al., 2020) is a freely available database of commercially available compounds developed by the Department of Pharmaceutical Chemistry at the University of California, San Francisco (UCSF). One of the most recent versions, ZINC20 (Irwin et al., 2020), contains over 10 millions of drug-like compounds. The DrugBank (Wishart et al., 2018) is a freely available database that includes drug information, drug targets, 3D structure and metabolic pathways. The database contains about 11,000 small compounds. ChEMBL (Mendez et al., 2019) is an open-source database developed by the European Bioinformatics Institute (EMBL-EBI) in Cambridge (United Kingdom). It also contains structures from the U.S. Food and Drug Administration (FDA). Information about approved products (from the FDA Orange Book), including dosage information and administration routes, is also included in the database. Currently, the database contains about 1.9 million drug-like compounds. The NuBBE database (Saldívar-González et al., 2019) is a natural product library created in 2013 that aims to collect the chemical structural diversity of Brazilian natural biodiversity, resulting in an extraordinary curated source of 2,223 natural compounds. Finally, the approved drug library used in this work is a collection of compounds downloaded from Selleck Chemicals that are ready to be used for HTS. The 3,104 compounds in this library are approved by different regulatory agencies such as the FDA or the European Medicines Agency (EMA), among others.

**FIGURE 7:**
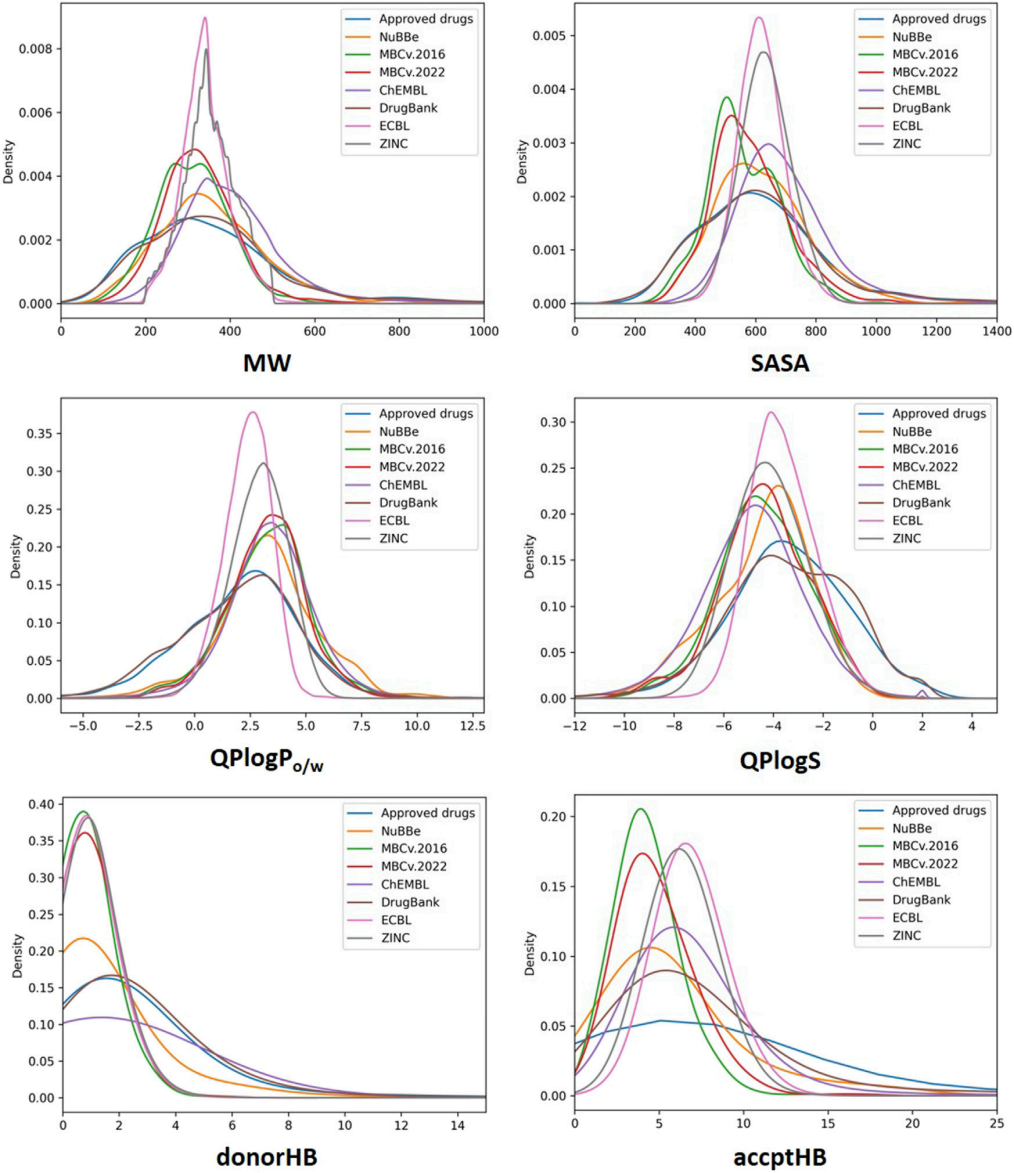
Probability density distribution of MW, SASA, QP logP_o/w_, QP logS, donorHB and accptHB properties for all the analyzed libraries.

Density distributions relative to the molecular weight, SASA, QP logP_o/w_, QP logS, donorHB (HBD) and accptHB (HBA) properties are reported in Figure 7. As shown, most of the compounds fall within the range of Lipinski’s rule of Five (that is, less than 500 Da) for MW with the MBC and ECBL having a slightly better fit among the analyzed databases. The solvent accessible surface area (SASA) for ECBL, ChEMBL and ZINC ranges between 400 and 900 Å^2^. A slightly shifter profile can be seen for MBC, DrugBank and the Approved drugs library, with SASA values from 200 to 800 Å^2^. Regarding NuBBE, the distribution seems to be an intermediate case compared to the previous ones, covering wider values from 200 to 1000 Å^2^ with a maximum population density close to that of the ECBL or ChEMBL. In the case of hydrogen-bond donor (donorHB) and acceptor (accptHB) properties, all the libraries apart from DrugBank, Approved drug library and ChEMBL for HBD, the vast majority agree with Lipinski’s rule of five and are in the range of 0.0–6.0 for HBD and 2.0–20.0 for HBA. This is likely due to the presence of small peptidomimetics and complex sugars in the previously cited libraries. Regarding lipophilicity (QP logP_o/w_), similar distributions in the range of −2.5 to 7.5 have been registered for all the libraries except for DrugBank and Approved drug library that have also some compounds with QP logP_o/w_ values below −2.5. Finally, compounds from MBC, ECBL, and ZINC are in the optimal range of solubility (−6.5 < QP logS <0.5). NuBBE and ChEMBL show a similar distribution with a small set of compounds with QP logS values lower than −6.5. However, the remaining compounds show an appropriate solubility profile. DrugBank and Approved drug library slightly deviate from the ideal range having a small number of compounds with QP logS values than 0.5.

## 4 Conclusion

The screening of quality-focused libraries could represent a way to efficiently provide a useful source of probes that help characterize the protein targets emerging from research studies. In this direction, efficient synthetic routes, availability, good characterization, suitable physicochemical and pharmacokinetic properties can really make the difference since they can contribute to rise the success rate and shorten the drug discovery process.

We here presented an updated version of our *in-house* MBC library which is a unique collection of small molecules with enriched drug-like properties and chemical diversity. From the first publication in 2016, the library has been constantly enriched with new compounds becoming 2.3 times bigger than the previous version with over 2,500 *ready-to-use* chemical compounds. To test its potential impact on drug discovery, the quality and variability of the chemical structures collected in the new version of the MBC library has been analyzed by using the QikProp module of Schrödinger and RDKit. As official partners and active collaborators of the EU-OPENSCREEN ERIC, we also presented and discussed the potentialities of the open-access European Chemical Biology Library (ECBL) that collect data from several countries in EU. Finally, a wider comparison with other well-known publicly available libraries has been provided and discussed. Results of this analysis remark the high quality in terms of structural diversity and drug-like properties of the MBC and ECBL, making them suitable reservoirs of hits for drug discovery.

## Data Availability

The datasets presented in this study can be found in online repositories. The name of the repository and accession number can be found in the article/Supplementary Material.

## References

[B1] Baell J. B. Holloway G. A. (2010). New substructure filters for removal of pan assay interference compounds (PAINS) from screening libraries and for their exclusion in bioassays. J. Med. Chem. 53 (7), 2719–2740. 10.1021/jm901137j 20131845

[B2] Bajorath J. (2002). Integration of virtual and high-throughput screening. Nat. Rev. Drug Discov. 1 (11), 882–894. 10.1038/nrd941 12415248

[B3] Bajusz D. Rácz A. Héberger K. (2015). Why is Tanimoto index an appropriate choice for fingerprint-based similarity calculations? J. Cheminform 7, 20. 10.1186/s13321-015-0069-3 26052348 PMC4456712

[B4] Balakin K. V. Tkachenko S. E. Kiselyov A. S. Savchuk N. P. (2006). Focused chemistry from annotated libraries. Drug Discov. Today Technol. 3 (4), 397–403. 10.1016/j.ddtec.2006.12.006

[B5] Bemis G. W. Murcko M. A. (1996). The properties of known drugs. 1. molecular frameworks. J. Med. Chem. 39 (15), 2887–2893. 10.1021/jm9602928 8709122

[B6] Bento A. P. Hersey A. Félix E. Landrum G. Gaulton A. Atkinson F. (2020). An open source chemical structure curation pipeline using RDKit. J. Cheminform 12 (1), 51. 10.1186/s13321-020-00456-1 33431044 PMC7458899

[B7] Bergström C. A. S. Larsson P. (2018). Computational prediction of drug solubility in water-based systems: qualitative and quantitative approaches used in the current drug discovery and development setting. Int. J. Pharm. 540 (1-2), 185–193. 10.1016/j.ijpharm.2018.01.044 29421301 PMC5861307

[B8] Blaschke T. Miljković F. Bajorath J. (2019). Prediction of different classes of promiscuous and nonpromiscuous compounds using machine learning and nearest neighbor analysis. ACS Omega 4 (4), 6883–6890. 10.1021/acsomega.9b00492

[B9] Brennecke P. Rasina D. Aubi O. Herzog K. Landskron J. Cautain B. (2019). EU-OPENSCREEN: a novel collaborative approach to facilitate chemical biology. SLAS Discov. 24 (3), 398–413. 10.1177/2472555218816276 30616481 PMC6764006

[B10] Frank R. (2014). EU-OPENSCREEN-a European infrastructure of open screening platforms for chemical biology. ACS Chem. Biol. 9 (4), 853–854. 10.1021/cb500189k 24742390

[B11] Garcia-Rubia A. Lasala F. Ginex T. Morales-Tenorio M. Olal C. Heung M. (2023). N'-Phenylacetohydrazide derivatives as potent Ebola virus entry inhibitors with an improved pharmacokinetic profile. J. Med. Chem. 66 (8), 5465–5483. 10.1021/acs.jmedchem.2c01785 37021830 PMC10150359

[B12] Gerry C. J. Schreiber S. L. (2018). Chemical probes and drug leads from advances in synthetic planning and methodology. Nat. Rev. Drug Discov. 17 (5), 333–352. 10.1038/nrd.2018.53 29651105 PMC6707071

[B13] Ghose A. K. Viswanadhan V. N. Wendoloski J. J. (1999). A knowledge-based approach in designing combinatorial or medicinal chemistry libraries for drug discovery. 1. a qualitative and quantitative characterization of known drug databases. J. Comb. Chem. 1 (1), 55–68. 10.1021/cc9800071 10746014

[B14] Ginex T. Vazquez J. Gilbert E. Herrero E. Luque F. J. (2019). Lipophilicity in drug design: an overview of lipophilicity descriptors in 3D-QSAR studies. Future Med. Chem. 11 (10), 1177–1193. 10.4155/fmc-2018-0435 30799643

[B15] Gong Z. Hu G. Li Q. Liu Z. Wang F. Zhang X. (2017). Compound libraries: recent advances and their applications in drug discovery. Curr. Drug Discov. Technol. 14 (4), 216–228. 10.2174/1570163814666170425155154 28443514

[B16] Graff D. E. Shakhnovich E. I. Coley C. W. (2021). Accelerating high-throughput virtual screening through molecular pool-based active learning. Chem. Sci. 12 (22), 7866–7881. 10.1039/d0sc06805e 34168840 PMC8188596

[B17] Horvath D. Lisurek M. Rupp B. Kühne R. Specker E. von Kries J. (2014). Design of a general-purpose European compound screening library for EU-OPENSCREEN. ChemMedChem 9 (10), 2309–2326. 10.1002/cmdc.201402126 25044981

[B18] Hu Y. Stumpfe D. Bajorath J. (2016). Computational exploration of molecular scaffolds in medicinal chemistry. J. Med. Chem. 59 (9), 4062–4076. 10.1021/acs.jmedchem.5b01746 26840095

[B19] Irwin J. J. Tang K. G. Young J. Dandarchuluun C. Wong B. R. Khurelbaatar M. (2020). ZINC20-A free ultralarge-scale chemical database for ligand discovery. J. Chem. Inf. Model. 60 (12), 6065–6073. 10.1021/acs.jcim.0c00675 33118813 PMC8284596

[B20] Jimonet P. Jäger R. (2004). Strategies for designing GPCR-focused libraries and screening sets. Curr. Opin. Drug Discov. Devel 7 (3), 325–333. 15216936

[B21] Kéri G. Székelyhidi Z. Bánhegyi P. Varga Z. Hegymegi-Barakonyi B. Szántai-Kis C. (2005). Drug discovery in the kinase inhibitory field using the Nested Chemical Library technology. Assay. Drug Dev. Technol. 3 (5), 543–551. 10.1089/adt.2005.3.543 16305311

[B22] Kim K. H. Kim N. D. Seong B. L. (2010). Pharmacophore-based virtual screening: a review of recent applications. Expert Opin. Drug Discov. 5 (3), 205–222. 10.1517/17460441003592072 22823018

[B23] Kuzikov M. Costanzi E. Reinshagen J. Esposito F. Vangeel L. Wolf M. (2021). Identification of inhibitors of SARS-CoV-2 3CL-Pro enzymatic activity using a small molecule *in vitro* repurposing screen. ACS Pharmacol. Transl. Sci. 4 (3), 1096–1110. 10.1021/acsptsci.0c00216 35287429 PMC7986981

[B24] Landrum G. (2016). RDKit: open-source cheminformatics software. Available at: https://www.rdkit.org/

[B25] Lasala F. Garcia-Rubia A. Requena C. Galindo I. Cuesta-Geijo M. A. Garcia-Dorival I. (2021). Identification of potential inhibitors of protein-protein interaction useful to fight against Ebola and other highly pathogenic viruses. Antivir. Res. 186, 105011. 10.1016/j.antiviral.2021.105011 33428961 PMC7833471

[B26] Lionta E. Spyrou G. Vassilatis D. K. Cournia Z. (2014). Structure-based virtual screening for drug discovery: principles, applications and recent advances. Curr. Top. Med. Chem. 14 (16), 1923–1938. 10.2174/1568026614666140929124445 25262799 PMC4443793

[B27] Lipinski C. A. (2004). Lead- and drug-like compounds: the rule-of-five revolution. Drug Discov. Today Technol. 1 (4), 337–341. 10.1016/j.ddtec.2004.11.007 24981612

[B28] Lipinski C. A. Lombardo F. Dominy B. W. Feeney P. J. (2001). Experimental and computational approaches to estimate solubility and permeability in drug discovery and development settings. Adv. Drug Deliv. Rev. 46 (1-3), 3–26. 10.1016/s0169-409x(00)00129-0 11259830

[B29] López-Vallejo F. Giulianotti M. A. Houghten R. A. Medina-Franco J. L. (2012). Expanding the medicinally relevant chemical space with compound libraries. Drug Discov. Today 17 (13-14), 718–726. 10.1016/j.drudis.2012.04.001 22515962

[B30] Maestro I. Madruga E. Boya P. Martínez A. (2023). Identification of a new structural family of SGK1 inhibitors as potential neuroprotective agents. J. Enzyme Inhib. Med. Chem. 38 (1), 2153841. 10.1080/14756366.2022.2153841 36637025 PMC9848319

[B31] Martínez-González L. Rodríguez-Cueto C. Cabezudo D. Bartolomé F. Andrés-Benito P. Ferrer I. (2020). Motor neuron preservation and decrease of *in vivo* TDP-43 phosphorylation by protein CK-1δ kinase inhibitor treatment. Sci. Rep. 10 (1), 4449. 10.1038/s41598-020-61265-y 32157143 PMC7064575

[B32] Mayr L. M. Bojanic D. (2009). Novel trends in high-throughput screening. Curr. Opin. Pharmacol. 9 (5), 580–588. 10.1016/j.coph.2009.08.004 19775937

[B33] Mendez D. Gaulton A. Bento A. P. Chambers J. De Veij M. Félix E. (2019). ChEMBL: towards direct deposition of bioassay data. Nucleic Acids Res. 47 (D1), D930–D940. 10.1093/nar/gky1075 30398643 PMC6323927

[B34] Neves B. J. Braga R. C. Melo-Filho C. C. Moreira-Filho J. T. Muratov E. N. Andrade C. H. (2018). QSAR-based virtual screening: advances and applications in drug discovery. Front. Pharmacol. 9, 1275. 10.3389/fphar.2018.01275 30524275 PMC6262347

[B35] Neves B. J. Mottin M. Moreira-Filho J. T. Sousa B. K. d. P. Mendonca S. S. Andrade C. H. (2021). “Chapter 4 - best practices for docking-based virtual screening,” in Molecular docking for computer-aided drug design. Editor Coumar M. S. (China: Academic Press), 75–98.

[B36] Nicolaou C. A. Watson I. A. Hu H. Wang J. (2016). The proximal Lilly collection: mapping, exploring and exploiting feasible chemical space. J. Chem. Inf. Model. 56 (7), 1253–1266. 10.1021/acs.jcim.6b00173 27286472

[B37] Rojas-Prats E. Martinez-Gonzalez L. Gonzalo-Consuegra C. Liachko N. F. Perez C. Ramirez D. (2021). Targeting nuclear protein tdp-43 by cell division cycle kinase 7 inhibitors: a new therapeutic approach for amyotrophic lateral sclerosis. Eur. J. Med. Chem. 210, 112968. 10.1016/j.ejmech.2020.112968 33139113

[B38] Salado I. G. Redondo M. Bello M. L. Perez C. Liachko N. F. Kraemer B. C. (2014). Protein kinase CK-1 inhibitors as new potential drugs for amyotrophic lateral sclerosis. J. Med. Chem. 57 (6), 2755–2772. 10.1021/jm500065f 24592867 PMC3969104

[B39] Saldívar-González F. I. Valli M. Andricopulo A. D. da Silva Bolzani V. Medina-Franco J. L. (2019). Chemical space and diversity of the NuBBE database: a chemoinformatic characterization. J. Chem. Inf. Model. 59 (1), 74–85. 10.1021/acs.jcim.8b00619 30508485

[B40] Sankara Rao A. Durga Bhavani S. Sobha Rani T. Raju S B. Narahari Sastry G. (2011). "Study of diversity and similarity of large chemical databases using Tanimoto measure", in: Computer Networks and Intelligent Computing, eds. Venugopal K. R. Patnaik L. M.: Germany, Springer Berlin Heidelberg), 40–50.

[B41] Schrödinger Release (2022). Schrödinger Release 2022-2. New York, NY: QikProp, Schrödinger, LLC.

[B42] Schrödinger (2022). Schrödinger Release 2022-2. New York, NY: LigPrep, Schrödinger, LLC.

[B43] Schuller M. Correy G. J. Gahbauer S. Fearon D. Wu T. Díaz R. E. (2021). Fragment binding to the Nsp3 macrodomain of SARS-CoV-2 identified through crystallographic screening and computational docking. Sci. Adv. 7 (16), eabf8711. 10.1126/sciadv.abf8711 33853786 PMC8046379

[B44] Sebastián-Pérez V. Roca C. Awale M. Reymond J. L. Martinez A. Gil C. (2017). Medicinal and Biological Chemistry (MBC) library: an efficient source of new hits. J. Chem. Inf. Model. 57 (9), 2143–2151. 10.1021/acs.jcim.7b00401 28813151

[B45] Sperandio O. Reynès C. H. Camproux A. C. Villoutreix B. O. (2010). Rationalizing the chemical space of protein-protein interaction inhibitors. Drug Discov. Today 15 (5-6), 220–229. 10.1016/j.drudis.2009.11.007 19969101

[B46] Stork C. Embruch G. Šícho M. de Bruyn Kops C. Chen Y. Svozil D. (2020). Nerdd: a web portal providing access to *in silico* tools for drug discovery. Bioinformatics 36 (4), 1291–1292. 10.1093/bioinformatics/btz695 32077475

[B47] Stork C. Mathai N. Kirchmair J. (2021). Computational prediction of frequent hitters in target-based and cell-based assays. Artif. Intell. Life Sci. 1, 100007. 10.1016/j.ailsci.2021.100007

[B48] Subramaniam S. Mehrotra M. Gupta D. (2008). Virtual high throughput screening (vHTS)-a perspective. Bioinformation 3 (1), 14–17. 10.6026/97320630003014 19052660 PMC2586130

[B49] Tanrikulu Y. Krüger B. Proschak E. (2013). The holistic integration of virtual screening in drug discovery. Drug Discov. Today 18 (7-8), 358–364. 10.1016/j.drudis.2013.01.007 23340112

[B50] Veber D. F. Johnson S. R. Cheng H. Y. Smith B. R. Ward K. W. Kopple K. D. (2002). Molecular properties that influence the oral bioavailability of drug candidates. J. Med. Chem. 45 (12), 2615–2623. 10.1021/jm020017n 12036371

[B51] Wishart D. S. Feunang Y. D. Guo A. C. Lo E. J. Marcu A. Grant J. R. (2018). DrugBank 5.0: a major update to the DrugBank database for 2018. Nucleic Acids Res. 46 (D1), D1074–D1082. 10.1093/nar/gkx1037 29126136 PMC5753335

[B52] Xia Z. Yan A. (2017). Computational models for the classification of mPGES-1 inhibitors with fingerprint descriptors. Mol. Divers 21 (3), 661–675. 10.1007/s11030-017-9743-x 28484935

