# Supplementary material for: MBC and ECBL libraries: outstanding tools for drug discovery
Source: Front Pharmacol. 2023 Aug 11;14:1244317. doi: 10.3389/fphar.2023.1244317 (PMC10457160; doi:10.3389/fphar.2023.1244317)
Supplement: Supplementary file 1 [file DataSheet2.pdf]

Supplementary Material

MBC and ECBL Libraries: outstanding tools for drug discovery

Tiziana Ginex,[1,†] Enrique Madruga,[1,2] Ana Martinez,[1,2] and Carmen Gil,[1,*]

[1] Centro de Investigaciones Biológicas "Margarita Salas" (CIB-CSIC), Ramiro de Maeztu 9, 28040 Madrid, Spain

[2] Centro de Investigación Biomédica en Red en Enfermedades Neurodegenerativas (CIBERNED), Instituto de Salud Carlos III

† Present address:

Dr. Tiziana Ginex — Pharmacelera, Parc Científic de Barcelona (PCB), Baldiri Reixac 4-8, 08028 Barcelona, Spain; E-mail: tiziana.ginex@pharmacelera.com

*** Correspondence:**

Dr. Carmen Gil; E-mail: carmen.gil@csic.es

All the material and data produced for this study along with the python scripts used to reproduce all the graphics can be found at https://doi.org/10.5281/zenodo.8212104. The following content is included: List of the 44 descriptors calculated with QikProp (DOCX format); Distribution (%) of the first 50 most populated Murcko scaffolds for MBC v.2016, MBC v.2022 and ECBL reported in **Figure 3** (EXCEL format); Distribution (count) of the first 94 common Murcko scaffolds for MBC v.2016, MBC v.2022 and ECBL reported in **Figure 5** (EXCEL format); QikProp properties (CSV format) and SMILES codes (SMI format) for all the analyzed DBs; Python script to generate the 2D plots in **Figure 3**; Python script to run and generate the Tanimoto similarity plots in **Figure 4**; Python script to run kernel density analysis reported in **Figure 7**; Python scripts to calculate the Ghose and Veber filters reported in **Table 1**.

List of principal descriptors and properties calculated with QikProp. A comprehensive list can be found at QikProp manual (http://gohom.win/ManualHom/Schrodinger/Schrodinger_2015-2_docs/qikprop/qikprop_user_manual.pdf)

**Principal Descriptors**	**Description**	**Range**
Molecular Weight	Molecular weight of the molecule.	130.0 / 725.0
Dipole Moment	Computed dipole moment of the molecule.	1.0 / 12.5
Total SASA	Total solvent accessible surface area (SASA) in square angstroms using a probe with a 1.4 Å radius.	300.0 / 1000.0
Hydrophobic SASA	Hydrophobic component of the SASA (saturated carbon and attached hydrogen).	0.0 / 750.0
Hydrophilic SASA	Hydrophilic component of the SASA (SASA on N, O, H on heteroatoms, carbonyl C).	7.0 / 330.0
Carbon Pi SASA	π (carbon and attached hydrogen) component of the SASA.	0.0 / 450.0
Weakly Polar SASA	Weakly polar component of the SASA (halogens, P, and S).	0.0 / 175.0
Volume	Total solvent-accessible volume in cubic angstroms using a probe with a 1.4 Å radius.	500.0 – 2000.0
PSA	Van der Waals surface area of polar nitrogen and oxygen atoms and carbonyl carbon atoms.	7.0 – 200.0
No. of Rotatable Bonds	Number of non-trivial (not CX3), non-hindered (not alkene, amide, small ring) rotatable bonds.	0 – 15
Donor - Hydrogen Bonds	Estimated number of hydrogen bonds that would be donated by the solute to water molecules in an aqueous solution. Values are averages taken over a number of configurations, so they can be non-integer.	0.0 – 6.0
Acceptor - Hydrogen Bonds	Estimated number of hydrogen bonds that would be accepted by the solute from water molecules in an aqueous solution. Values are averages taken over a number of configurations, so they can be non-integer.	2.0 – 20.0
Globularity	Globularity descriptor, $(4\pi r^2)/(SASA)$ where r is the radius of a sphere with a volume equal to the molecular volume. Globularity is 1.0 for a spherical molecule.	0.75 / 0.95
Ionization Potential	PM3 calculated ionization potential (negative of HOMO energy).	7.9 – 10.5
Electron Affinity	PM3 calculated electron affinity (negative of LUMO energy).	–0.9 – 1.7
QP Polarizability	Pedicted polarizability in cubic angstroms.	13.0 – 70.0
QPlogPC16	Predicted hexadecane/gas partition coefficient.	4.0 – 18.0
QPlogPoct	Predicted octanol/gas partition coefficient.	8.0 – 35.0
QPlogPw	Predicted water/gas partition coefficient.	4.0 – 45.0
QPlogPo/w	Predicted octanol/water partition coefficient.	–2.0 – 6.5
QPlogS	Predicted aqueous solubility, log S. S in mol dm^{-3} is the concentration of the solute in a saturated solution that is in equilibrium with the crystalline solid.	–6.5 – 0.5
CIQPlogS	Conformation-independent predicted aqueous solubility, log S. S in mol dm^{-3} is the concentration of the solute in a saturated solution that is in equilibrium with the crystalline solid.	–6.5 – 0.5
QPlogKhsa	Prediction of binding to human serum albumin.	–1.5 – 1.5
QPlogBB	Predicted brain/blood partition coefficient.	–3.0 – 1.2
N°. of Primary Metabolites	Number of likely metabolic reactions.	1.0 / 8.0
CNS	Predicted central nervous system activity on a –2 (inactive) to +2 (active) scale.	–2 (inactive), +2 (active)
QPlogHERG	Predicted IC_{50} value for blockage of HERG K^+ channels.	concern below –5
QPPCaco	Predicted apparent Caco-2 cell permeability in nm/sec. Caco2 cells are a model for the gut-blood barrier. QikProp predictions are for non-active transport.	<25 poor, >500 great
QPPMDCK	Predicted apparent MDCK cell permeability in nm/sec. MDCK cells are considered to be a good mimic for the bloodbrain barrier. QikProp predictions are for non-active transport.	<25 poor, >500 great

QPlogKp	Predicted skin permeability, log Kp.	–8.0 – –1.0
Jm	Predicted maximum transdermal transport rate, Kp × MW × S (μg cm^{-2} hr^{-1}). Kp and S are obtained from the aqueous solubility and skin permeability, QPlogKp and QplogS.	
RuleOfFive	Number of violations of Lipinski's rule of five. The rules are: mol_MW < 500, QPlogPo/w < 5, donorHB ≤ 5, accptHB ≤ 10. Compounds that satisfy these rules are considered druglike.	maximum is 4
RuleOfThree	Number of violations of Jorgensen's rule of three. The three rules are: QPlogS > -5.7, QP PCaco > 22 nm/s, # Primary Metabolites < 7. Compounds with fewer (and preferably no) violations of these rules are more likely to be orally available.	maximum is 3
PercentHumanOralAbsorption	Predicted human oral absorption on 0 to 100% scale. The prediction is based on a quantitative multiple linear regression model.	>80% is high, <25% is poor
